# Urine Biomarkers of Risk in the Molecular Etiology of Breast Cancer

**DOI:** 10.4137/bcbcr.s2112

**Published:** 2009-01-06

**Authors:** Nilesh W. Gaikwad, Li Yang, Sandhya Pruthi, James N. Ingle, Nicole Sandhu, Eleanor G. Rogan, Ercole L. Cavalieri

**Affiliations:** 1 Eppley Institute for Research in Cancer and Allied Diseases, University of Nebraska Medical Center, 986805 Nebraska Medical Center, Omaha, NE 68198-6805; 2 Breast Diagnostic Clinic, Internal Medicine, Mayo Clinic, 200 First Street, SW, Rochester, MN 55905; 3 Department of Medical Oncology, Mayo Clinic, 200 First Street, SW, Rochester, MN 55905; 4 Department of Environmental, Agricultural and Occupational Health, College of Public Health, University of Nebraska Medical Center, 985110 Nebraska Medical Center, Omaha, NE 68198-5110

**Keywords:** breast cancer risk, depurinating estrogen-DNA adducts, urinary estrogen biomarkers, balance in estrogen metabolism

## Abstract

Endogenous estrogens can be bio-activated to endogenous carcinogens via formation of estrogen quinones. Estrogen-3,4-quinones react with DNA to form mutagenic depurinating estrogen-DNA adducts. The carcinogenicity of endogenous estrogens is related to unbalanced estrogen metabolism leading to excess estrogen quinones and formation of depurinating DNA adducts. The present studies were initiated to confirm that relatively high levels of depurinating estrogen-DNA adducts are present in women at high risk for breast cancer or diagnosed with the disease. These adducts may be biomarkers for early detection of breast cancer risk. The estrogen metabolites, conjugates and depurinating DNA adducts were identified and quantified by using ultraperformance liquid chromatography/tandem mass spectrometry to analyze urine samples from 40 healthy control women, 40 high-risk women and 40 women with newly diagnosed breast cancer. Estrogen metabolism was shifted from protective methoxylation and conjugation pathways in healthy control women towards activating pathways leading to formation of depurinating DNA adducts in women at high risk or with breast cancer. These results support the hypothesis that breast cancer is initiated by mutations derived from depurination of estrogen-DNA adducts. Therefore, relative levels of depurinating estrogen-DNA adducts could become biomarkers for early detection of breast cancer risk and aid in determining preventive strategies.

## Introduction

Estrogens, which contain a benzene ring, can become endogenous carcinogens. Three lines of evidence support this concept. Exposure to estrogens is a risk factor for increased incidence of breast cancer.^1^,^2^ Other evidence derives from the similar reaction of benzene *ortho*-quinone and estrogen *ortho*-quinones with nucleophiles. In fact, these two compounds react with DNA to form analogous depurinating DNA adducts.^3^ Exposure to benzene is a risk factor for lymphoma and leukemia.^4^,^5^ Finally, the *ortho*-quinones of benzene and estradiol (E_2_) induce similar hyperproliferation of human peripheral blood mononuclear cells.^6^

**Figure 1.**
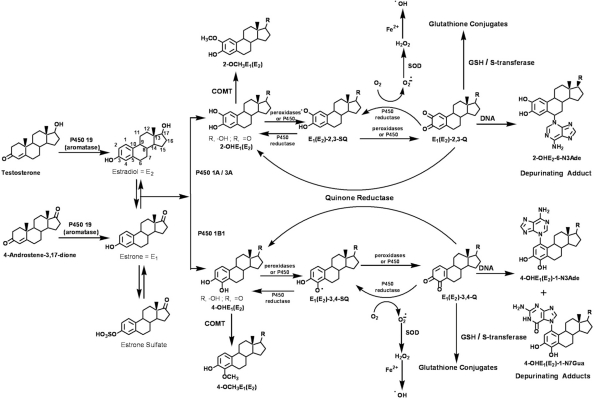
Biosynthesis and metabolic activation of the estrogens E_1_ and E_2_. One of the major metabolic pathways of E_1_ and E_2_ leads to 2- and 4-catechol derivatives, which further oxidize to yield the corresponding reactive quinones. The quinones can react with DNA to form depurinating DNA adducts. In the deactivation pathway, which operates in parallel, the catechol derivatives are methylated to form methoxy catechol estrogens; in addition, the quinones are reduced by quinone reductase, as well as being conjugated with GSH, and, are thus rendered harmless. A shift in the apparent balance between these activating and deactivating pathways towards formation of depurinating DNA adducts could lead to the initiation of breast cancer.

In an earlier study, we have shown that in healthy control women (n = 46) whose estrogen metabolism is considered balanced, the level of estrogen-DNA adducts in urine is low and/or the levels of estrogen catechol metabolites, and thiol and methyl conjugates are high. In contrast, in high-risk women (n = 12) and those with breast cancer (n = 17), the unbalanced estrogen metabolism is reflected in higher levels of estrogen-DNA adducts in urine and/or lower levels of estrogen metabolites and conjugates.^7^ It is this imbalance in estrogen metabolism, leading to relatively high levels of estrogen-DNA adducts, that is the determinant of breast cancer initiation (Fig. 1). In principle, environmental, genetic, as well as dietary, factors can unbalance the equilibrium between the activating and deactivating pathways of estrogen metabolism.

Once released from DNA, the predominant depurinating estrogen-DNA adducts, 4-hydroxyestrone (estradiol)-1-N3Adenine [4-OHE_1_(E_2_)-1-N3Ade] and 4-hydroxyestrone(estradiol)-1-N7Guanine [4-OHE_1_(E_2_)-1-N7Gua]^8^–^10^ are shed from cells into the bloodstream and, eventually, excreted in urine.^7^ Release of the depurinating adducts generates apurinic sites in the DNA, which in turn induce mutations. It is thought that critical mutations generated by specific DNA damage can result in abnormal cell proliferation leading to cancer.^11^–^15^ The transforming activity of E_2_ and 4-OHE_2_ has been observed in human breast epithelial (MCF-10 F) cells, which do not contain estrogen receptor-α, and are not affected by the presence of an anti-estrogen.^16^–^19^ E6 mouse mammary cells were also transformed by treatment with E_2_-3,4-quinone (Q).^20^ Furthermore, 4-OHE_1_(E_2_) are carcinogenic in the Syrian golden hamster and CD-1 mouse.^21^–^24^ All of these studies support the hypothesis that estrogens initiate cancer through their genotoxicity.

In light of our earlier findings,^7^ we wanted to validate them with a larger number of participants. We conducted a cross-sectional study in which 40 estrogen metabolites, conjugates and depurinating DNA adducts were analyzed in urine samples from 40 healthy control women, 40 women at high risk for breast cancer based on a Gail Model score ≥1.66%, and 40 women with newly diagnosed Stage 0, 1 and 2 breast carcinoma (*in-situ* and invasive carcinoma), all recruited at the Mayo Clinic. The results presented here provide further support that the ratios of depurinating DNA adducts to their respective estrogen metabolites and conjugates were significantly associated with risk status.

## Materials and Methods

### Materials

Phenyl solid phase extraction (SPE) cartridges were purchased from Varian (Palo Alto, CA). Androstenedione 1, testosterone 2, E_1_-sulfate 3, E_2_ 4, E_1_ 5, 2-OHE_2_ 6, 2-OHE_1_ 7, 16α-OHE_2_ 10, 16α-OHE_1_ 11, 2-OCH_3_E_2_ 12, 2-OCH_3_E_1_ 13, 4-OCH_3_E_2_ 14, 4-OCH_3_E_1_ 15, 2-OH-3-OCH_3_E_2_ 16 and 2-OH-3-OCH_3_E_1_ 17 were purchased from Steraloids, Inc. (Newport, RI). 4-OHE_2_ 8 and 4-OHE_1_ 9 were synthesized as previously described.^25^ 2-OHE_2_-1-glutathione (SG) 18, 2-OHE_2_-4-SG 19, 2-OHE_1_-1-SG 20, 2-OHE_1_-4-SG 21, 2-OHE_2_-(1 + 4)-cysteine (Cys) 22, 2-OHE_1_-1-Cys 23, 2-OHE_1_-4-Cys 24, 2-OHE_2_-1-*N*-acetylcysteine (NAcCys) 25, 2-OHE_2_-4-NAcCys 26, 2-OHE_1_-1-NAcCys 27, 2-OHE_1_-4-NAcCys 28, 4-OHE_2_-2-SG 29, 4-OHE_1_-2-SG 30, 4-OHE_2_-2-Cys 31, 4-OHE_1_-2-Cys 32, 4-OHE_2_-2-NAcCys 33 and 4-OHE_1_-2-NAcCys 34 were synthesized by using the procedure of Cao et al.^26^ 4-OHE_2_-1-N7Gua 35, 4-OHE_1_-1-N7Gua 36, 4-OHE_2_-1-N3Ade 37, 4-OHE_1_-1-N3Ade 38, 2-OHE_2_-6-N3Ade 39 and 2-OHE_1_-6-N3Ade 40 were synthesized by following reported methods.^9^,^10^,^27^ All solvents were HPLC grade and all other chemicals used were of the highest grade available.

### Study population

Spot urine samples were collected from 120 women at the Breast Diagnostic Clinic and Oncology Breast Clinic of the Mayo Clinic, Rochester, Minnesota. Women were recruited between October 2006 and December 2007 and their ages ranged between 23 and 84 years. Healthy control women: range, 23–63; median, 44.5 years; high-risk women: range, 35–76; median, 57.3 years; women with breast cancer: range, 35–84; median, 57.8 years.

The 40 healthy control women had not received a diagnosis of breast cancer at the time of their urine collection and had a calculated Gail Model score of <1.66%. Among the 40 high-risk women, their Gail Model scores were 1.67%–11.7%. The Gail Model is a validated tool developed by the National Cancer Institute 28 that takes into account a woman’s age, number of breast biopsies and prior history of atypical hyperplasia, age at menarche, and number of affected first degree relatives. It calculates both a 5-year risk and lifetime risk. A 5-year Gail Model score of ≥1.66% is considered high risk. The final 40 participants were diagnosed with Stage 0, 1 and 2 breast cancer within 30 days of providing the urine sample. None of the participants received estrogen-containing treatment for at least 3 months prior to providing a urine sample.

The protocol was approved by the Mayo Clinic and UNMC Institutional Review Boards. Signed consents included authorization to collect and bank urine samples and collect demographic and clinical information.

### Sample collection

A spot urine sample of about 50 ml was collected from each participant and 1 mg/ml ascorbic acid was added to prevent oxidation of the catechol moieties in the various estrogen compounds. The urine samples were aliquoted, frozen, and four 10-ml aliquots were transferred to the Eppley Institute, UNMC, on dry ice and stored at −80 °C until analysis. Thus, each analytical sample was thawed only once prior to analysis.

### Solid phase extraction of urine

The SPE method development and validation were described earlier.^7^ Briefly, after adjusting 2-ml aliquots of urine to pH 7, they were loaded onto the 100-mg phenyl cartridges pre-conditioned with methanol and the loading buffer, 10 mM ammonium formate, pH 7. The cartridges were washed with the loading buffer, and then the compounds of interest were eluted from the cartridge by using an elution buffer, methanol:10 mM ammonium formate, pH 7 (90:10), with 1% acetic acid. The eluates from both the experimental and control samples were concentrated and subjected to ultra-performance liquid chromatography/tandem mass spectrometry (UPLC/MS-MS) analysis.

We previously found that the treatment of urine with glucuronidase/sulfatase led to significant increases (10–20 fold) in the levels of E_1_ and E_2_, however, the levels of estrogen metabolites, conjugates and adducts changed marginally and in many cases decreased because of the incubation for 8 h at 37 °C. Hence, to avoid artifacts and errors that are introduced by maintaining the urine samples at 37 °C for 8 h, we carried out all the analyses without treating the samples with glucuronidase/sulfatase. For this reason, the reported levels of E_1_ and E_2_ are 10–15 times less than the total values.^7^

### UPLC/MS-MS analysis of urine samples

All experiments were performed on a Waters (Milford, MA) Quattro Micro triple quadrupole mass spectrometer by using electrospray ionization (ESI) in positive ion (PI) and negative ion (NI) mode, with an ESI-MS capillary voltage of 3.0 kV, an extractor cone voltage of 2 V, and a detector voltage of 650 V. Desolvation and cone gas flow were maintained at 400 and 60 l/h respectively. Desolvation temperature and source temperature were set to 200 and 100 °C, respectively. For all the studies, a methanol:water (1:1) mixture with 0.1% formic acid was used as the carrier solution. The parent and daughter ion data obtained for all standard compounds were used to generate the multiple reaction monitoring (MRM) method for UPLC/MS-MS operation.^7^

UPLC/MS analyses of estrogen-related compounds (Table 1) in urine extracts were carried out with a Waters Acquity UPLC system connected with the high performance Quattro Micro triple quadrupole mass spectrometer. Analytical separations on the UPLC system were conducted using an Acquity UPLC BEH C18 1.7 μm column (1 × 100 mm) at a flow rate of 0.15 ml/min. The gradient started with 80% A (0.1% formic acid in H_2_O) and 20% B (0.1% formic acid in acetonitrile), changed to 79% A over 4 min, followed by a 6-min linear gradient to 45% A, resulting in a total separation time of 10 min. The levels of compounds were normalized to the concentration of creatinine (Cr). The elutions from the UPLC column were introduced to the Quattro Micro mass spectrometer. Resulting data were processed by using QuanLynx software (Waters) to quantify the estrogen metabolites, conjugates and DNA adducts.^7^

## Statistical Analyses

### Statistical methods

Median values were calculated for all estrogen compounds obtained from healthy control, high risk and breast cancer groups. Ratios were compared for healthy control vs. high risk and for healthy control vs. breast cancer using one-way ANOVA. Additional post hoc analysis was done for multiple comparisons using Dunn’s method. All the statistics and *p*-values were calculated using GraphPad Prism software V 4.03 (GraphPad Software, La Jolla, CA).

## Results and Discussion

Using the SPE/UPLC/MS-MS methodology recently developed by our laboratory,^7^ we have analyzed urine samples from various groups of women for 40 estrogen-related compounds (Fig. 1). This analysis resulted in data reporting the concentration of each of the 40 compounds (Table 1). Furthermore, we calculated the ratio of depurinating N3Ade and N7Gua adducts to the sum of their respective estrogen metabolites and conjugates in the urine samples because this ratio reflects the degree of imbalance in estrogen metabolism that can lead to cancer initiation (Fig. 1).

**Figure 2.**
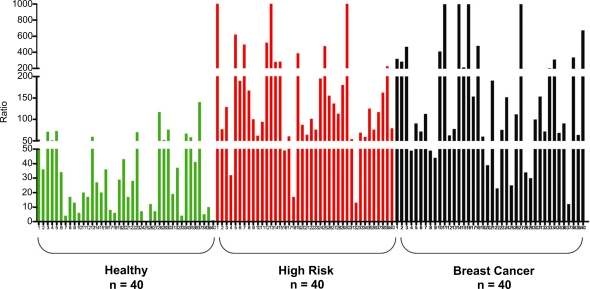
Depurinating estrogen-DNA adducts in the urine of healthy control women, women at high risk for breast cancer, and women with breast cancer. The ordinate of this bar graph corresponds to the ratio of depurinating DNA adducts divided by their respective estrogen metabolites, and thiol and methyl conjugates:
$$\left[\frac{{4-\text{OHE}}_{1}\;({\text{E}}_{2})-1-\text{N}3\text{Ade}+4{-\text{OHE}}_{1}\;({\text{E}}_{2})-1-\text{N}7\text{Gua}}{4-\text{catechol}\;\text{estrogens}+4-\text{catechol}\;\text{estrogen}\;\text{conjugates}}+\frac{{2-\text{OHE}}_{1}\;({\text{E}}_{2})-6-\text{N}3\text{Ade}}{2-\text{catechol}\;\text{estrogens}+2-\text{catechol}\;\text{estrogen}\;\text{conjugates}}\right]\times 1000,$$i.e.
$$\left[\frac{\text{No}.37+38+35+36}{\text{No}.8+9+14+15+29\;\text{through}\;34}+\frac{\text{No}.39+40}{\text{No}.6+7+12+13+16\;\text{through}\;28}\right]\times 1000\;(\text{see}\;\text{Table}\;1\;\text{for}\;\text{the}\;\text{numbers}).$$

The ratios in healthy control women are generally low (Fig. 2). In contrast, high ratios of these adducts to estrogen metabolites and conjugates were observed in urine from high-risk women (Gail Model score ≥1.66%) and women with breast carcinoma (Fig. 2). In general, the value obtained from the high-risk women and women with breast carcinoma derives from the ratio between a high level of adducts and low levels of metabolites and conjugates. In some women, however, the level of adducts was not particularly high, but the levels of metabolites and conjugates were very low, suggesting that a substantial proportion of the metabolites was converted to adducts. In addition, the average contribution of the 2-OHE_1_(E_2_)-6-N3Ade adducts to the total ratio was found to be insignificant, whereas the predominant contribution was from the 4-OHE_1_(E_2_)-1-N3Ade and 4-OHE_1_(E_2_)-1-N7Gua adducts.^7^ The healthy control women had a lower median age than the high-risk and breast cancer women. Multiple linear regression comparison of the ratios between the three groups after adjusting for age indicated that the median ratio for the healthy control women group is still significantly different from the median ratios for the high-risk and breast cancer groups. The median ratios for the high-risk and breast cancer groups are not significantly different. The results obtained in this study are similar to those obtained by analyzing serum samples collected from the same women.^29^

**Figure 3.**
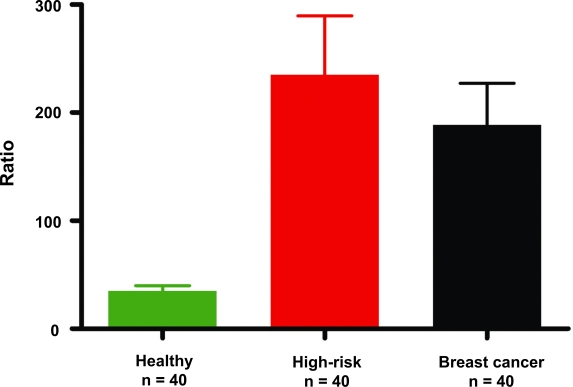
Mean ratios of depurinating estrogen-DNA adducts to their respective metabolites and conjugates. The mean sum of the ratios for control women was significantly lower than those for the high-risk women (p < 0.001) and women with breast cancer (p < 0.001). The mean sums of the ratios for high-risk women and women with breast cancer were not significantly different.

As seen in our earlier study, analysis of the ratio using one-way ANOVA reveals a significant difference between the high-risk and breast cancer groups compared to the healthy control group (p < 0.001) (Fig. 3). Additional post hoc analysis using a Dunn’s correction for multiple comparisons revealed significantly higher means for high-risk subjects [mean 232, standard deviation (SD) 340] compared with healthy controls (mean 34.7, SD 31.6, p < 0.001) and for breast cancer patients (mean 188, SD 235, p < 0.001) compared to controls (Fig. 3). The mean for subjects known to be at high risk was not significantly different from that of the breast cancer group (Fig. 3).

**Figure 4.**
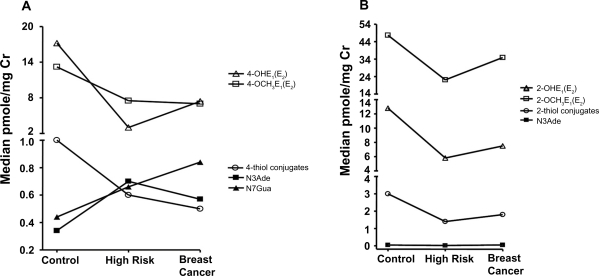
**A**) Urinary median levels of 4-OHE_1_(E_2_), 4-OCH_3_E_1_(E_2_) and 4-OHE_1_(E_2_)-thiol conjugates, as well as 4-OHE_1_(E_2_)-1-N3Ade and 4-OHE_1_(E_2_)-1-N7Gua adducts in urine samples from 40 healthy control women, 40 high-risk women and 40 women with breast cancer. **B**) Urinary median levels of 2-OHE_1_(E_2_), 2-OCH_3_E_1_(E_2_) and 2-OHE_1_(E_2_)-thiol conjugates, as well as the 2-OHE_1_(E_2_)-6-N3Ade adducts, in urine samples from 40 healthy control women, 40 high-risk women and 40 women with breast cancer.

Each sample was analyzed in triplicate and the mean values were calculated for all 40 compounds (Table 1). Finally, the median values obtained for selected compounds using the 40 samples in the three different groups were graphed (Fig. 4). As seen in our earlier study, the median 4-OHE_1_(E_2_), 4-OCH_3_E_1_(E_2_) and 4-OHE_1_(E_2_)-thiol conjugate (GSH, Cys, NAcCys) values were higher for healthy controls compared with high-risk subjects and breast cancer cases (Fig. 4A). In contrast, the median 4-OHE_1_(E_2_)-1-N3Ade and 4-OHE_1_(E_2_)-1-N7Gua values were lower for healthy controls compared with high-risk subjects and breast cancer cases (Fig. 4A). Compared with breast cancer cases and high-risk subjects, the median 2-OHE_1_(E_2_), 2-OCH_3_E_1_(E_2_), and 2-OHE_1_(E_2_)-thiol conjugate values were higher for healthy controls (Fig. 4B), while the median 2-OHE_1_(E_2_)-6-N3Ade values did not show any trend. The rest of the metabolites (Table 1) are not shown because they are not included in the calculation of the ratios or they did not show any significant trend in the three groups.

In conclusion, these results confirm our previous finding of high ratios of depurinating estrogen-DNA adducts to their corresponding metabolites and conjugates in urine samples from both high-risk women and women with breast cancer, compared to lower ratios in healthy control women.^7^ This finding is consistent with the hypothesis that formation of estrogen-DNA adducts is the first critical step in the initiation of breast cancer. These results suggest that a urine assay may provide a screening biomarker for early detection of breast cancer risk. However, further larger studies are required to know how far in advance this assay would predict the development of a clinically or mammographically detectable tumor.

## Conclusion

The ratio of depurinating estrogen-DNA adducts to their metabolites and conjugates may be a urine biomarker to quantify an individual’s risk and determine appropriate risk reducing strategies. Another use of the urine assay could be to assess the ability of natural antioxidants to balance estrogen activation and deactivation, thereby reducing the risk of developing breast cancer.

## Figures and Tables

**Table 1.** Representative metabolic profile of a urine sample obtained from a high-risk woman.

**No.**	**Compound**	**pmol/mg Cr mean, n = 3**	**Total pmol/mg Cr**
1	Androstenedione	0.7 ± 0.1^a^	**0.7**
2	Testosterone	0.0 ± 0.0	**0.0**
3	E_1_-Sulfate	3.3 ± 0.8	**3.3**
4	E_2_	0.6 ± 0.3	**5.4**
5	E_1_	4.8 ± 0.8	
6	2-OHE_2_	1.6 ± 1.5	**1.6**
7	2-OHE_1_	0.0 ± 0.0	
8	4-OHE_2_	0.0 ± 0.0	**0.0**
9	4-OHE_1_	0.0 ± 0.0	
10	16α-OHE_2_	6.4 ± 3.2	**93.9**
11	16α-OHE_1_	87.5 ± 25.9	
12	2-OCH_3_E_2_	4.5 ± 0.8	**35.5**
13	2-OCH_3_E_1_	31.0 ± 10.5	
14	4-OCH_3_E_2_	2.0 ± 0.8	**8.3**
15	4-OCH_3_E_1_	6.3 ± 4.5	
16	2-OH-3-OCH_3_E_2_	0.0 ± 0.0	**0.0**
17	2-OH-3-OCH_3_E_1_	0.0 ± 0.0	
18	2-OHE_2_-1-SG	0.0 ± 0.0	**1.4**
19	2-OCH_2_-4-SG	0.0 ± 0.0	
20	2-OHE_1_-1-SG	0.5 ± 0.3	
21	2-OHE_1_-4-SG	0.5 ± 0.3	
22	2-OHE_2_-1+4-Cys	0.2 ± 0.2	
23	2-OHE_1_-1-Cys	0.0 ± 0.0	
24	2-OHE_1_-4-Cys	0.0 ± 0.0	
25	2-OHE_2_-1-NAcCys	0.0 ± 0.1	
26	2-OHE_2_-4-NAcCys	0.0 ± 0.1	
27	2-OHE_1_-1-NAcCys	0.0 ± 0.0	
28	2-OHE_1_-4-NAcCys	0.0 ± 0.0	
29	4-OHE_2_-2-SG	0.3 ± 0.2	**0.8**
30	4-OHE_1_-2-SG	0.4 ± 0.3	
31	4-OHE_2_-2-Cys	0.0 ± 0.0	
32	4-OHE_1_-2-Cys	0.0 ± 0.0	
33	4-OHE_2_-2-NAcCys	0.0 ± 0.0	
34	4-OHE_1_-2-NAcCys	0.0 ± 0.0	
35	4-OHE_2_-1-N7Gua	0.01 ± 0.01	**4.63**
36	4-OHE_1_-1-N7Gua	4.62 ± 2.39	
37	4-OHE_2_-1-N3Ade	0.96 ± 0.08	**1.02**
38	4-OHE_1_-1-N3Ade	0.06 ± 0.04	
39	2-OHE_2_-6-N3Ade	0.03 ± 0.03	**0.03**
40	2-OHE_1_-6-N3Ade	0.0 ± 0.0	

a Mean ± standard deviation.

## Abbreviations

Cr: creatinine;
Cys: cysteine;
ESI: electrospray ionization;
E_1_(E_2_)-Q: estrone(estradiol)-quinone;
GSH: glutathione;
4-OHE_1_(E_2_): 4-hydroxyestrone (estradiol);
4-OHE_1_(E_2_)-1-N3Ade: 4-hydroxyestrone (estradiol)-1-N3Adenine;
4-OHE_1_(E_2_)-1-N7Gua: 4-hydroxyestrone(estradiol)-1-N7Guanine;
2-OHE_1_(E_2_)-6-N3Ade: 2-hydroxyestrone (estradiol)-6-N3Adenine;
MRM: multiple reaction monitoring;
NAcCys: *N*-acetylcysteine;
NI: negative ion;
PI: positive ion;
SD: standard deviation;
SPE: solid-phase extraction;
UPLC/MS-MS: ultraperformance liquid chromatography/tandem mass spectrometry.
